# Astaxanthin and meclizine extend lifespan in UM-HET3 male mice; fisetin, SG1002 (hydrogen sulfide donor), dimethyl fumarate, mycophenolic acid, and 4-phenylbutyrate do not significantly affect lifespan in either sex at the doses and schedules used

**DOI:** 10.1007/s11357-023-01011-0

**Published:** 2023-12-02

**Authors:** David E. Harrison, Randy Strong, Peter Reifsnyder, Nadia Rosenthal, Ron Korstanje, Elizabeth Fernandez, Kevin Flurkey, Brett C. Ginsburg, Meredith D. Murrell, Martin A. Javors, Marisa Lopez-Cruzan, James F. Nelson, Bradley J. Willcox, Richard Allsopp, David M. Watumull, David G. Watumull, Gino Cortopassi, James L. Kirkland, Tamar Tchkonia, Young Geun Choi, Matthew J. Yousefzadeh, Paul D. Robbins, James R. Mitchell, Murat Acar, Ethan A. Sarnoski, Michael R. Bene, Adam Salmon, Navasuja Kumar, Richard A. Miller

**Affiliations:** 1 The Jackson Laboratory, 600 Main Street, Bar Harbor, ME 04609 USA; 2 Barshop Institute for Longevity and Aging Studies, The University of Texas Health Science Center, San Antonio, TX USA; 3 Education, and Clinical Center, Geriatric Research, San Antonio, TX USA; 4 Research Service, South Texas Veterans Health Care System, San Antonio, TX USA; 5 Department of Pharmacology, The University of Texas Health Science Center, San Antonio, TX USA; 6 Department of Psychiatry, The University of Texas Health Science Center, San Antonio, TX USA; 7 Department of Physiology, The University of Texas Health Sciences Center, San Antonio, TX USA; 8 John A. Burns School of Medicine, University of Hawai‘I at Mānoa, Honolulu, HI USA; 9 Cardax, Honolulu, HI USA; 10 AX3 Life, Honolulu, HI USA; 11 Department of Molecular Biosciences, University of California, Davis, CA USA; 12 Mayo Clinic, Rochester, MN USA; 13 University of Minnesota, Minneapolis, MN USA; 14 Harvard School of Public Health, Boston, MA USA; 15 Department of Basic Medical Sciences, School of Medicine, Koç University, 34450 Istanbul, Turkey; 16 Department of Internal Medicine, Yale University, New Haven, CT USA; 17 Department of Molecular Medicine, The University of Texas Health Sciences Center, San Antonio, TX USA; 18 Department of Internal Medicine, University of Michigan, Ann Arbor, MI USA; 19 Department of Pathology and Geriatrics Center, University of Michigan, Ann Arbor, MI USA

**Keywords:** Lifespan, Heterogeneous mice, Fisetin (Fis), SG1002, Astaxanthin (Asta), Meclizine (Mec), Dimethyl Fumarate (DMF), Mycophenolic acid (MPA), 4-Phenylbutyrate (PBA)

## Abstract

In genetically heterogeneous (UM-HET3) mice produced by the CByB6F1 × C3D2F1 cross, the Nrf2 activator astaxanthin (Asta) extended the median male lifespan by 12% (*p* = 0.003, log-rank test), while meclizine (Mec), an mTORC1 inhibitor, extended the male lifespan by 8% (*p* = 0.03). Asta was fed at 1840 ± 520 (9) ppm and Mec at 544 ± 48 (9) ppm, stated as mean ± SE (*n*) of independent diet preparations. Both were started at 12 months of age. The 90th percentile lifespan for both treatments was extended in absolute value by 6% in males, but neither was significant by the Wang–Allison test. Five other new agents were also tested as follows: fisetin, SG1002 (hydrogen sulfide donor), dimethyl fumarate, mycophenolic acid, and 4-phenylbutyrate. None of these increased lifespan significantly at the dose and method of administration tested in either sex. Amounts of dimethyl fumarate in the diet averaged 35% of the target dose, which may explain the absence of lifespan effects. Body weight was not significantly affected in males by any of the test agents. Late life weights were lower in females fed Asta and Mec, but lifespan was not significantly affected in these females. The male-specific lifespan benefits from Asta and Mec may provide insights into sex-specific aspects of aging.

## Introduction

Each year, the Interventions Testing Program (ITP; http://www.nia.nih.gov/research/dab/interventions-testing-program-itp) tests the effects of a variety of compounds on lifespan in a genetically heterogeneous mouse model (UM-HET3), the first generation offspring of the CByB6F1 × C3D2F1 cross, to produce a diverse heterogeneous population that is reproducible across time and place [1]. ITP studies are conducted simultaneously at The University of Texas San Antonio (UTSA), University of Michigan (UM), and The Jackson Laboratory (JAX) in Bar Harbor, ME. Details of the ITP design have been published [2–12]. Interventions that are found, in an initial experiment, to increase lifespan are then subsequently tested for effects on physiological and pathological changes with age. The data in this paper come from initial survival cohorts and focus on lifespan, which integrates a number of biological and other effects that influence risk of mortality. The pathology at death in UM-HET3 mice has been reported by [13], using groups of 136 and 208 virgin females, 268 multiparous females, and 117 and 157 virgin males.

The interventions for the present study were chosen for the following reasons:

(a) Astaxanthin (Asta) is a naturally occurring xanthophyll carotenoid [14, 15] that is an efficient Nrf2 activator [16], with potent antioxidant activity [17, 18], broad health applications [19], and excellent safety [20]. Asta is distributed systemically [21] and incorporated into cellular membranes, where it spans and stabilizes the lipid bilayer and reduces lipid peroxidation [22]. The molecular structure of Asta allows efficient scavenging of free radicals both at the surface and inside of the membrane [23]. Asta localizes in mitochondria and protects against mitochondrial dysfunction [24, 25]. It has anti-inflammatory properties [26, 27], showing equivalent efficacy to prednisolone in an animal model.

Sorrenti et al. [28] reviewed the geroprotective mechanisms of Asta, highlighting its regulation of FOXO3, Nrf2, Sirt1, and Klotho. They also demonstrated that Asta significantly increased the FOXO3 gene expression in the heart tissue of mice. In addition, the influence of Asta on autophagy via modulation of AMPK (a direct upstream regulator of mTOR), PI3K/Akt, and MAPK (JNK and p38) signaling pathways was reviewed [29].

Asta extended the lifespan in various models. In *Caenorhabditis elegans*, Asta increased the lifespan 16–30% in wild type and *age-1* worms, but not in *daf-16* knock-out worms [30]. Furthermore, Asta extended the lifespan in *C. elegans* by inducing autophagy through the IIS and TOR signaling pathways [31]. In *Saccharomyces cerevisiae* yeast, Asta increased the lifespan by decreasing oxidative stress and apoptosis [32]. Asta also extended the lifespan of *D. melanogaster* flies under oxidative stress [33, 34]. The present ITP study is the first evaluation of Asta in a mammalian lifespan model, so the target dose of 4000 ppm in the diet is based on chronic mammalian studies other than lifespan. Despite the fact that the average diet contained 1840 ppm Asta (only 46% of the target), median lifespans of male UM-HET3 mice were significantly improved.

(b) Meclizine (Mec) has been used as an antihistamine, anti-nausea, and anti-vertigo drug; it also has central anticholinergic actions [35, 36]. Also, it binds mTOR and inhibits mTORC1 [37]. This may be relevant for aging, since another mTORC1-inhibitor, rapamycin, significantly extends lifespan in both male and female UM-HET3 mice [5, 9, 10]. In addition, inhibition of mTORC1 by drugs or genetics extends lifespan and health in yeast, flies, worms, and mice [reviewed by 38]. mTORC1 inhibitors are thus of great interest in the biology of aging. Rapamycin has damaging side effects, such as increasing diabetes and suppressing immune responses and red blood cell production [38, 39], so a TORC1 inhibitor without such side effects that increases mammalian lifespan is of clinical interest.

Allen et al. [37] screened 1600 drugs for TOR binding and found that 3-ring piperazines including Mec specifically inhibit mTORC1 but not mTORC2. Mec has been used since 1955 as an anti-nausea or vertigo treatment, with minimal side effects [35]. Finally, Mec may benefit injury or degeneration in the brain [40, 41]. The target dose of 800 ppm Mec in the diet is based on doses that benefit mice in previous studies [42]. Despite the fact that the average diet contained 544 ppm Mec (only 68% of the target), median lifespans of male HET3 mice were significantly improved.

(c) Senescent cells have been reported as important mediators of the pathophysiology of aging, and senolytics like fisetin (Fis) may play important roles in mediating their effects. Zhu et al. [43] showed that Fis removed senescent cells from human senescent endothelial but not preadipocyte (mesenchymal) cell cultures. Yousefzadeh et al. [44] treated naturally aged or progeroid mutant mice with Fis and found that it reduced cells with senescent markers; for example, C57BL/6 mice at 23 ± 1 months old were given Fis or vehicle for 5 days by oral gavage. Three days later, in inguinal fat, controls averaged 8% SA-β-gal^+^ cells, while Fis-treated fat had 2%. They also fed (B6 X FVB)F1 hybrid mice 500 ppm Fis from 19 months of age and found that the median lifespan was 27 months in controls and 30 months in Fis-treated, with 3 of 8 treated mice outliving all 8 controls. In aged (B6 X FVB) F1 mice, Fis feeding reduced amylase and alanine aminotransferase in serum, suggesting improved pancreatic and liver function. Age-related lesions in the brain, kidney, liver, lung, and forepaw tissue sections were reduced by Fis feeding. Finally, in fat, spleen, liver, and kidney, senescence (p16^Ink4a^ and p21^Cip1^) and senescence-associated secretory phenotype markers (IL-1β, IL-6, IL-10, TNF-α, CXCL2, MCP-1, and PAI-1) were reduced in mice fed Fis [44].

We elected to use 600 ppm Fis, starting at 20 months of age, since senescent cells are present in potentially harmful quantities starting at that age in inbred C57BL/6 J or F1 mice, based on [44]. We fed either continuously or for 3 days every 2 weeks, as detailed in the discussion.

(d) SG1002 is designed to slowly release hydrogen sulfide (H_2_S), which has numerous beneficial biological effects, including lifespan extension in the invertebrate nematode *C. elegans* [45]. Furthermore, H_2_S production is stimulated by dietary restriction (DR), causing Hine et al. [46] to suggest that H_2_S may to some degree cause some of the benefits of DR. The target dose of 240 ppm SG1002 in the diet is based on doses that reduced hepatic ischemia reperfusion injury in mice [47].

(e) Dimethyl fumarate (DMF) is a Nrf2 stimulator currently used to treat human psoriasis and relapsing multiple sclerosis [48]. It also increases the production of mitochondria in humans and mice and is the first drug shown to do this in human beings [49]. It also reduces inflammation in microglia [50]. Nrf stimulation, the anti-inflammatory effects and the benefits of reducing or reversing loss of mitochondria function with age, causes the ITP to test effects of DMF on lifespan. The target dose of 120 ppm DMF in the diet is based on doses that increased the mitochondrial number in mice (48). The fact that the average diet contained 42 ppm DMF (only 35% of the target) may help explain why lifespans were not affected.

(f) Mycophenolic acid (MPA) inhibits inosine monophosphate dehydrogenase (IMD), thus inhibiting guanosine monophosphate (GMP) synthesis [51]. Like rapamycin, it was FDA-approved to retard organ transplant rejection (under the brand name CellCept). It has also been used to treat autoimmune diseases [52] and increased the replication potential (“lifespan”) of *S. cerevisiae* [53]. Eickenberg et al. [54] showed that it inhibits B cell proliferation and plasma blast formation in people with lupus, thus inhibiting an autoimmune disease. In unpublished experiments by proposers, the target dose of 6.7 ppm MPA in the diet was estimated to achieve a minimum plasma concentration to provide useful effects.

(g) 4-Phenylbutyrate (PBA) increases lifespan in flies [55] and has major benefits in mouse models of neurodegenerative disease [56]. PBA also reduces inflammation and monocyte increase due to aging [57]. Since PBA benefits diseases of age, as well as reducing inflammation, the ITP tested whether it extends UM-HET3 mouse lifespan. The target dose of 1000 ppm PBA in the diet is in the high range of doses that benefited models of Parkinson disease [58].

## Results

Lifespan data for cohort 2018 (C2018) are shown separately from cohort 2019 (C2019), because the studies were started in different years (2018 or 2019). The cohorts used independent controls, and the statistics were done comparing the interventions tested in each cohort with the controls from the same cohort. Three of the six drugs were unexpectedly low in the actual diets fed for lifespan studies. In the protocol for food testing, for each diet preparation, UT tests 3 separate pellets, each at 3 separate locations (each end and the middle). These are far less variable than the different diet preparations for Asta, Mec, and DMF, the three that averaged unexpectedly low. Target doses of interventions, and the overall average dose of all preparations fed to the mice in lifespan studies, are given in the caption of Table 1. Averages for the amount of intervention in each diet preparation and the month and year of the preparation are given in the “Experimental procedures” section, under the “Test agents given to cohort 2018” section, and the “Test agents given to cohort 2019” section. The high variability from batch to batch and low average amounts of Asta, Mec and DMF illustrate the importance of measuring the actual amounts of test drugs in the diet. Failure to measure the amount of an intervention actually in the diet may lead to misinformation, because amounts in the diet sometimes vary greatly from what is intended.

**Table 1:** Effects of drugs on lifespans of UM-HET3 mice, pooled data

	Agent	*n*	Median	% for med	Log rank	p90	% for p90	Wang–Allison
Males								
2018	Cont_18	292	750	NA		1019	NA	
	Fis_Cyc	150	799	7	0.54	1011	− 1	0.74
	Fis_On	142	715	− 5	0.85	1016	0	1.00
	SG1002	153	764	2	0.62	1032	1	0.62
	Cont_19	298	817	NA		1092	NA	
2019	Asta	156	911	12	0.003	1159	6	0.19
	DMF_16	148	808	− 1	0.80	1082	− 1	0.62
	DMF_early	149	825	1	0.51	1135	4	0.32
	Mec	149	885	8	0.03	1156	6	0.14
	MPA	149	827	1	0.19	1147	5	0.19
	PBA	152	844	3	0.26	1127	3	0.25
	SG1002_2	150	814	0	0.79	1121	3	0.51
Females								
2018	Cont_18	286	876	NA		1081	NA	
	Fis_Cyc	134	871	− 1	0.75	1098	2	0.73
	Fis_On	144	883	1	0.37	1073	− 1	0.87
	SG1002	139	885	1	0.20	1122	4	0.50
	Cont_19	282	893	NA		1098	NA	
2019	Asta	132	924	3	0.64	1088	− 1	0.73
	DMF_16	136	885	− 1	0.20	1074	− 2	0.23
	DMF_early	135	896	0	0.97	1103	0	0.86
	Mec	136	917	3	0.40	1106	1	1.00
	MPA	133	882	− 1	0.05	1049	− 4	0.16
	PBA	135	879	− 2	0.01	1036	− 6	0.06
	SG1002_2	134	874	− 2	0.51	1071	− 2	0.30

*n* = number of mice tested; data from the 3 sites were pooled, about 1/3 of the mice were from each site. Agent: Test agent name, target dose in diet, measured percentage of target dose in diet ± SE (number of independent diet preparations tested), age test diet started (same for males and females): cohort 2018: controls (Cont_18); Fisetin (Fis), 600 ppm, 99% ± 9 (5). Twenty months, fed continuously (Fis_On) or a repeat of 3 days on and 11 days off (Fis_Cyc); SG1002 (hydrogen sulfide donor), 240 ppm, could not be measured, fed continuously from 16 months. Cohort 2019: controls (Cont_19); astaxanthin (Asta), 4000 ppm, 46% ± 13 (9), fed continuously from 12 months; dimethyl fumarate (DMF), 120 ppm, 35% ± 17 (6), fed continuously from 10 months (DMF_early) or 16 months (DMF_16); Meclizine (Mec), 800 ppm, 68% ± 6 (9), fed continuously from 12 months; mycophenolic acid (MPA), 6.7 ppm, 91% ± 7 (9), fed continuously from 9 mo.;

4-Phenylbutyrate (PBA), 1000 ppm, 107% ± 6 (9), fed continuously from 9 months; SG1002 (hydrogen sulfide donor), 240 ppm, could not be measured. Fed continuously from 5 to 9 months. Median lifespan: days–median ages; percent change calculated with respect to controls. *p*-value is equal to probability that lifespans are the same as the controls using two-tailed log-rank test on pooled data stratified by sites; “removed” mice were included as censored (see the “Experimental procedures” section). Lifespan at the 90th percentile: days = age at the 90th percentile, percent change–from control. Wang-Allison *p*-value is equal to probability that the proportion of live mice is the same in treated as in the control group at the 90th percentile age, evaluated by the procedure of Wang et al. (2004)

**Fig. 1:**
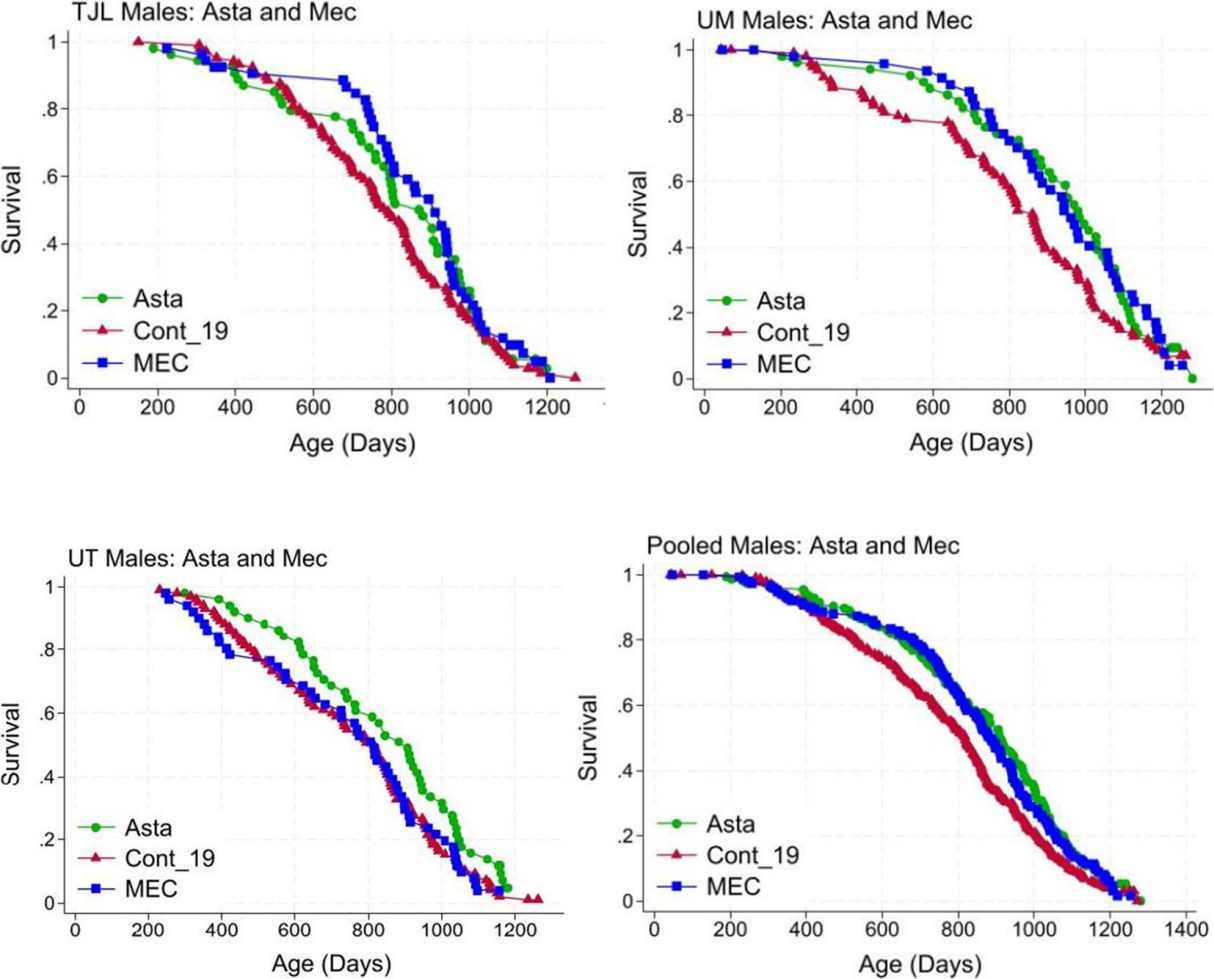
Lifespans of male HET3 mice fed Asta or MEC compared to controls

**Fig. 2:**
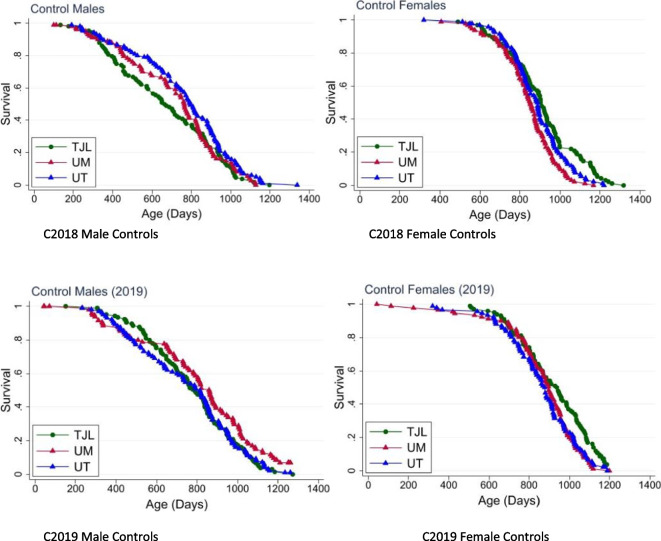
Site-specific lifespan curves for controls. Site specific lifespan data were more variable than usual

**Fig. 3:**
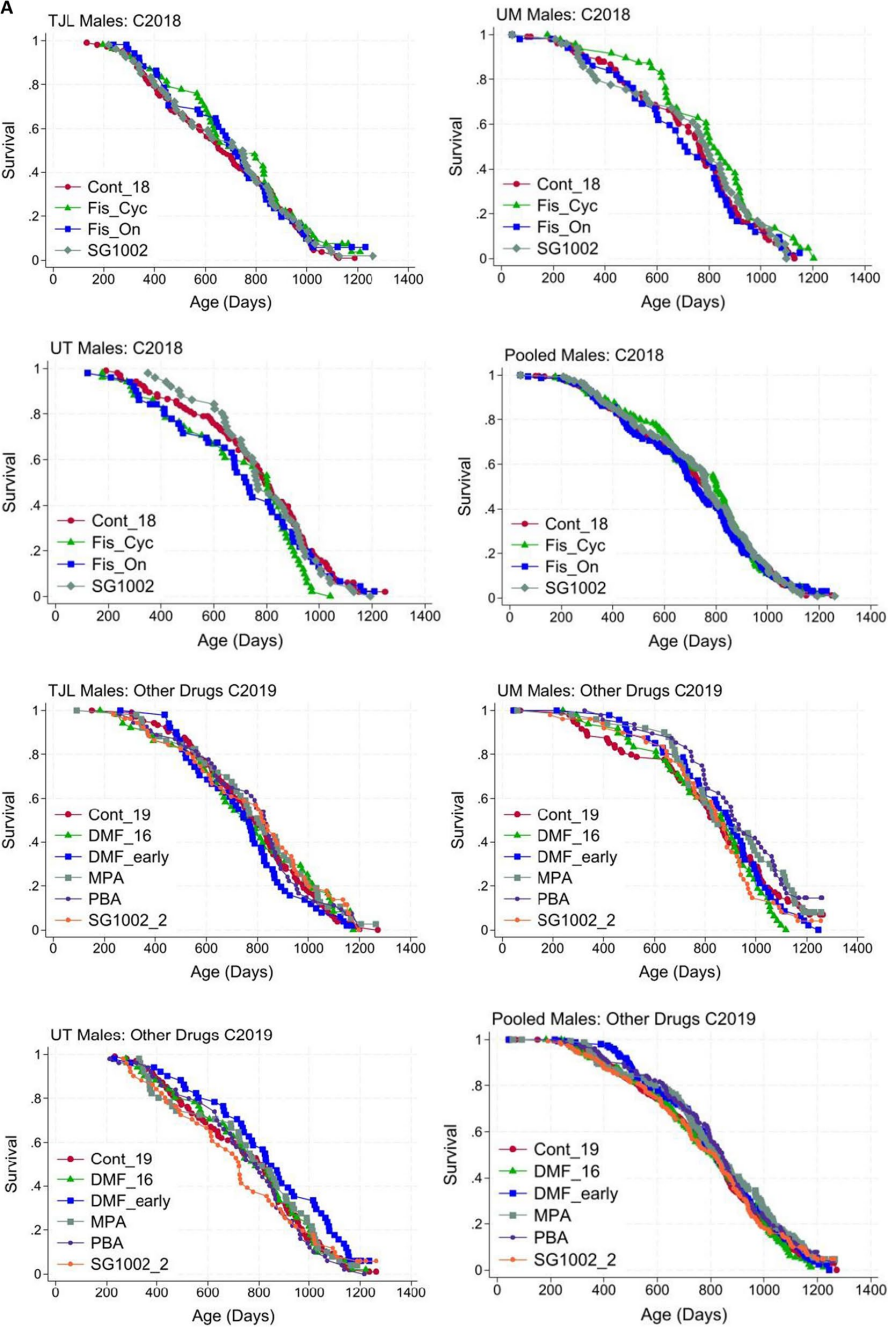

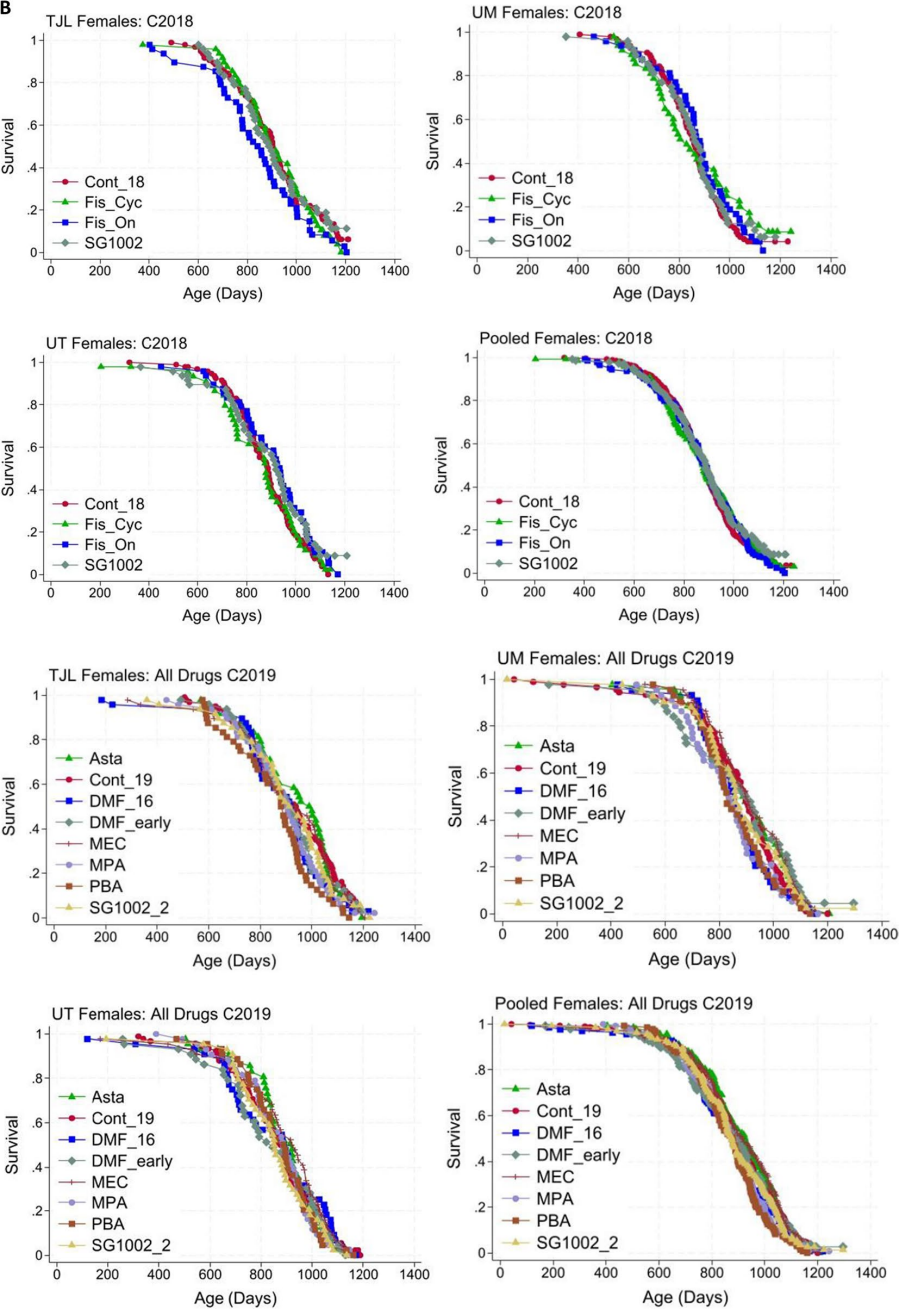
**A** Site-specific and pooled lifespan curves for drugs with no significant effects in HET3 males. **B** Site-specific and pooled lifespan curves for drugs with no significant effects in HET3 females

Table 1 shows that, compared to controls, UM-HET3 male mice fed Asta starting at 12 months of age had their median lifespan increased by 12%. The target amount of Asta in the diet was 4000 ppm, but actual amounts were 46% of that, SE ± 13, in 9 diet preparations. The log-rank statistic showed *p* = 0.003 with data pooled across all three sites. Age at the 90th percentile was increased by 6% in males, but our test for extreme lifespan, the Wang–Allison test, was not significant (*p* = 0.19). Looking at each site, Asta increased the median male lifespan to 14% at UM, 11% at JAX, and 11% at UT (Tables 2, 3, and 4). Males fed Mec from 12 months of age had their median lifespan increased 8%, and the effect was significant using the log-rank test at *p* = 0.03. The target amount of Mec in the diet was 800 ppm, but actual amounts were 68% of that, SE ± 6, in 9 diet preparations. Age at the 90th percentile was increased 6%, but the Wang–Allison test for extreme survival was not significant (*p* = 0.14; Table 1). Mec increased the median male lifespan to 11% at UM, 15% at JAX, and 0 at UT (Tables 2, 3, and 4). Lifespan curves for males given Asta, Mec, and controls are compared in Fig. 1 for each site and for pooled data and suggest that benefits diminished in the last 20% of the lifespan. Neither Asta nor Mec significantly altered the female lifespan (Tables 1, 2, 3, and 4; Figs. 2 and 3B). Testing levels in blood, 5 males had a Mec mean ± SD = 174 ± 77 ng/ml; 8 females had a Mec mean ± SD = 115 ± 100 ng/ml. The higher blood levels in males could explain why Mec extended the lifespan significantly in males only.

**Table 2:** Lifespans at the Jackson Laboratory

	Agent	*n*	Median	% for med	Log rank	p90	% for p90	Wang–Allison
Males								
2018	Cont_18	108	667			1009		
	Fis_Cyc	54	759	14	0.16	1034	2	0.27
	Fis_On	51	717	7	0.44	996	− 1	1.00
	SG1002	54	749	12	0.58	1003	− 1	0.80
	Cont_19	105	790	NA		1068	NA	
2019	Asta	54	875	11	0.270	1067	0	1.00
	DMF_16	49	756	− 4	0.65	1096	3	0.15
	DMF_early	51	765	− 3	0.38	1029	− 4	0.58
	Mec	51	911	15	0.06	1104	3	0.40
	MPA	48	803	2	0.40	1123	5	0.58
	PBA	54	830	5	0.53	1127	6	0.41
	SG1002_2	51	822	4	0.25	1141	7	0.16
Females								
2018	Cont_18	96	905			1167		
	Fis_Cyc	48	902	0	0.58	1112	− 5	0.37
	Fis_On	48	848	− 6	0.06	1063	− 9	0.37
	SG1002	48	892	− 1	0.89	1186	2	1.00
	Cont_19	100	939	NA		1144	NA	
2019	Asta	48	994	6	0.96	1116	− 2	0.55
	DMF_16	48	918	− 2	0.14	1075	− 6	0.55
	DMF_early	48	914	− 3	0.54	1123	− 2	0.55
	Mec	48	940	0	0.72	1137	− 1	0.77
	MPA	48	904	− 4	0.12	1103	− 4	0.09
	PBA	48	885	− 6	0.004	1066	− 7	0.04
	SG1002_2	48	928	− 1	0.54	1120	− 2	0.55

Same as Table 1. Except *n* = number of mice from each site

**Table 3:** Lifespans at University of Michigan

	Agent	*n*	Median	% for med	Log rank	p90	% for p90	Wang–Allison
Males								
2018	Cont_18	82	760			1014		
	Fis_Cyc	45	801	5	0.07	1123	11	0.54
	Fis_On	44	696	− 8	0.94	1069	5	0.77
	SG1002	48	789	4	0.50	1059	4	0.23
	Cont_19	94	861	NA		1178	NA	
2019	Asta	51	983	14	0.049	1193	1	0.78
	DMF_16	48	868	1	0.18	1055	− 10	0.02
	DMF early	47	894	4	0.76	1111	− 6	0.77
	Mec	47	960	11	0.08	1191	1	0.55
	MPA	50	842	− 2	0.37	1183	0	0.78
	PBA	48	923	7	0.07	1197	2	
	SG1002	48	858	0	0.45	1098	− 7	0.39
Females								
2018	Cont_18	96	852			1002		
	Fis_Cyc	42	798	− 6	0.14	1079	8	0.031
	Fis_On	48	882	4	0.11	1056	5	0.092
	SG1002	48	863	1	0.34	1079	8	1.00
	Cont_19	90	896	NA		1068	NA	
2019	Asta	44	892	0	0.38	1088	2	0.39
	DMF_16	44	852	− 5	0.34	1043	− 2	0.77
	DMF_early	44	905	1	0.29	1098	3	0.23
	Mec	44	901	1	0.24	1106	4	0.39
	MPA	42	832	− 7	0.18	1022	− 4	0.55
	PBA	43	827	− 8	0.27	1036	− 3	1.00
	SG1002_2	42	858	− 4	0.71	1076	1	0.77

Same as Table 1. Except *n* = number of mice from each site

**Table 4:** Lifespans at University of Texas

	Agent	*n*	Median	% for med	Log rank	p90	% for p90	Wang–Allison
Males								
2018	Cont_18	102	794			1047		
	Fis_Cyc	51	801	1	0.02	942	− 10	0.022
	Fis_On	47	721	− 9	0.68	1039	− 1	0.78
	SG1002	51	765	− 4	0.73	1005	− 4	0.58
	Cont_19	99	814	NA		1090	NA	
2019	Asta	51	905	11	0.030	1159	6	0.15
	DMF_16	51	785	− 4	0.73	1029	− 6	1.00
	DMF_early	51	847	4	0.03	1151	6	0.15
	Mec	51	815	0	0.97	1053	− 3	1.00
	MPA	51	810	0	0.60	1069	− 2	1.00
	PBA	50	789	− 3	0.65	1038	− 5	0.77
	SG1002_2	51	721	− 11	0.99	1100	1	0.58
Females								
2018	Cont_18	94	887			1068		
	Fis_Cyc	44	877	− 1	0.71	1082	1	0.77
	Fis_On	48	935	5	0.11	1131	6	0.27
	SG1002	43	918	3	0.15	1099	3	0.24
	Cont_19	92	878	NA		1076	NA	
2019	Asta	40	916	4	0.97	1042	− 3	0.55
	DMF_16	44	892	2	0.87	1083	1	0.77
	DMF_early	43	845	− 4	0.60	1060	− 1	1.00
	Mec	44	926	5	0.52	1081	0	0.77
	MPA	43	885	1	0.60	1018	− 5	0.39
	PBA	44	890	1	0.55	1028	− 4	0.23
	SG1002_2	44	851	− 3	0.37	1048	− 3	0.55

Same as Table 1. Except *n* = number of mice from each site

Site-specific statistics are given in Tables 2, 3, and 4. Both Asta and MEC significantly increase median male lifespan at JAX and UM, but only Asta at UT. When data are pooled from all three sites (Table 1), both Asta and MEC significantly increase median male lifespan. Conclusions are based on pooled data.

These data are pooled to give the controls for males and females in C2018 and C2019 in Table 1 and Fig. 3. Median lifespan, lifespan at the 90th percentile, and statistics relating control and intervention-fed mice at each site are given in Tables 2, 3, and 4.

Tables 2, 3, and 4 give statistics for site-specific lifespans. Doses and start times for the drugs are given in Table 1, which also gives the statistics for pooled lifespan comparisons. None of these interventions increased lifespans significantly. Conclusions are based on pooled data. Tables 2, 3, and 4 give statistics for site-specific lifespans. Doses and start times for the drugs are given in Table 1, which also gives the statistics for pooled lifespan comparisons. None of these interventions increased lifespans significantly. Conclusions are based on pooled data.

None of the other interventions in Table 1 significantly altered the male or female lifespan, except that 4-phenylbutyrate (PBA) reduced the pooled female lifespan by 2%, statistically significant (*p* = 0.01). The 90th percentile for PBA in females was reduced 6%, and this approached significance using the Wang–Allison test (*p* = 0.06). The target amount of DMF in the diet was 120 ppm, but actual amounts were 35% of that, SE ± 17, in 6 diet preparations. Using only a third of the target amount may explain why DMF did not significantly affect lifespans. Actual amounts of SG-1002 could not be measured with our equipment. The other interventions had the target amounts in the diets, so this does not explain why they failed to alter lifespans. Giving intervention–target amount, mean percent of target measured ± SE (number of diet preparations tested), the amounts were: Fisetin—600 ppm, 99% ± 9 (5); MPA—6.7 ppm, 91% ± 7 (9); and PBA—1000 ppm, 107% ± 6 (9).

Figure 2 shows the lifespan of control mice at each of the three sites for cohorts 2018 and 2019. For C2018 males, UM did not differ from either JAX or UT, but in that cohort, JAX males were short-lived compared to UT (*p* = 0.02). While female controls usually have a similar lifespan at all sites, in C2018, the female controls lived longest at JAX, next longest at UT, and shortest at UM: For JAX > UM, *p* < 0.0001; UT > UM, *p* = 0.02; JAX > UT, *p* = 0.04. These site-specific differences occurred despite the effort taken to use very similar husbandry procedures and suppliers at all three sites and illustrate the importance of replication at independent sites. For C2019, UM males were significantly longer lived than males at UT or JAX; UT and JAX males did not differ significantly, a pattern noted in nearly all previous ITP cohorts: For the C2019 male controls UM > JAX, *p* = 0.03; UM > UT, *p* = 0.03. C2019 female controls lived longest at JAX and were similar at UM and UT: JAX > UM, *p* = 0.006; JAX > UT, *p* = 0.01. In most previous years, the differences among sites in the survival of female controls were small and not statistically significant.

The site-specific and pooled lifespan curves for all the tested agents that did not significantly affect lifespan are shown in Fig. 3A (males) and Fig. 3B (females), with those for C2018 and C2019 shown separately. Two DMF groups were started at 10 or 16 months because the access panel and our Steering Committee felt this agent deserved testing at both ages. As noted above, amounts of DMF in the diets assayed were very low (“Experimental procedures”, Drugs given cohort 2019, c). This may help to explain the absence of a statistically significant lifespan effect.

Two SG1002 groups were used because at the mid-point of C2018, late-start SG1002 appeared effective, and we used an early start for this drug in C2019, as it might be even more effective. Unfortunately, the mid-point analysis was not replicated when the data were complete. The lifespan curves of the test agents that did not have statically significant effects on lifespan are very similar to the matched control data. As always, the absence of significant effects on lifespan may be due to the dose, schedule, or mouse model.

**Fig. 4:**
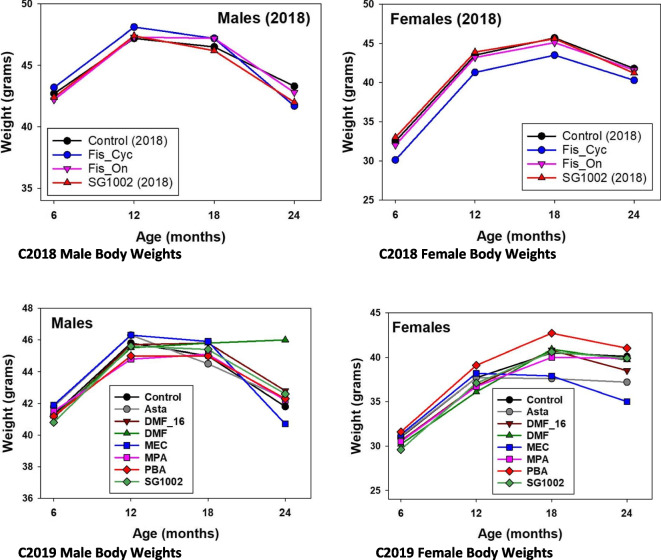
Body weights over the lifespan. Body weights for the mice in Table 1

Figure 4 shows body weights for the mice in Table 1. For C2018, the body weights did not differ significantly over 6–24 months in male treatment groups. Mice assigned to the Fis_Cyc treatment were, for unknown reasons, slightly but significantly heavier (males) or lighter (females) in weight compared to mice of the other three C2018 groups, but these differences were before treatment began and cannot be attributed to Fis. None of the C2018 groups differed statistically significantly in weight at ages 18 and 24 months. For C2019, there were no significant weight difference in males, and only males showed lifespan effects. Both Asta and Mec reduced body weights in females at 18 and 24 months of age, but these agents did not affect female lifespan in a statistically significant manner.

We used a one-way ANOVA to compare treatment groups for each age and sex. When differences were significant, we used a post hoc test (Sidak), adjusted for multiple comparisons, to test which groups differed from controls. For C2018 and C2019, there were no significant effects of drugs on male weights. For C2018 females, Fis Cyc had slightly reduced weights at 6 and 12 months, before Fis was fed. For C2019 females, weights at 6 months—W6—gave *p* = 0.07 before drugs were fed. W12: *p* = 0.03, but no drug-treated group differed significantly from controls. W18: *p* = 0.0001. Asta < control at *p* = 0.03. For MEC, *p* = 0.09. W24: *p* < 0.0001 with Asta < control, *p* = 0.04. MEC < control, *p* < 0.001.

**Fig. 5:**
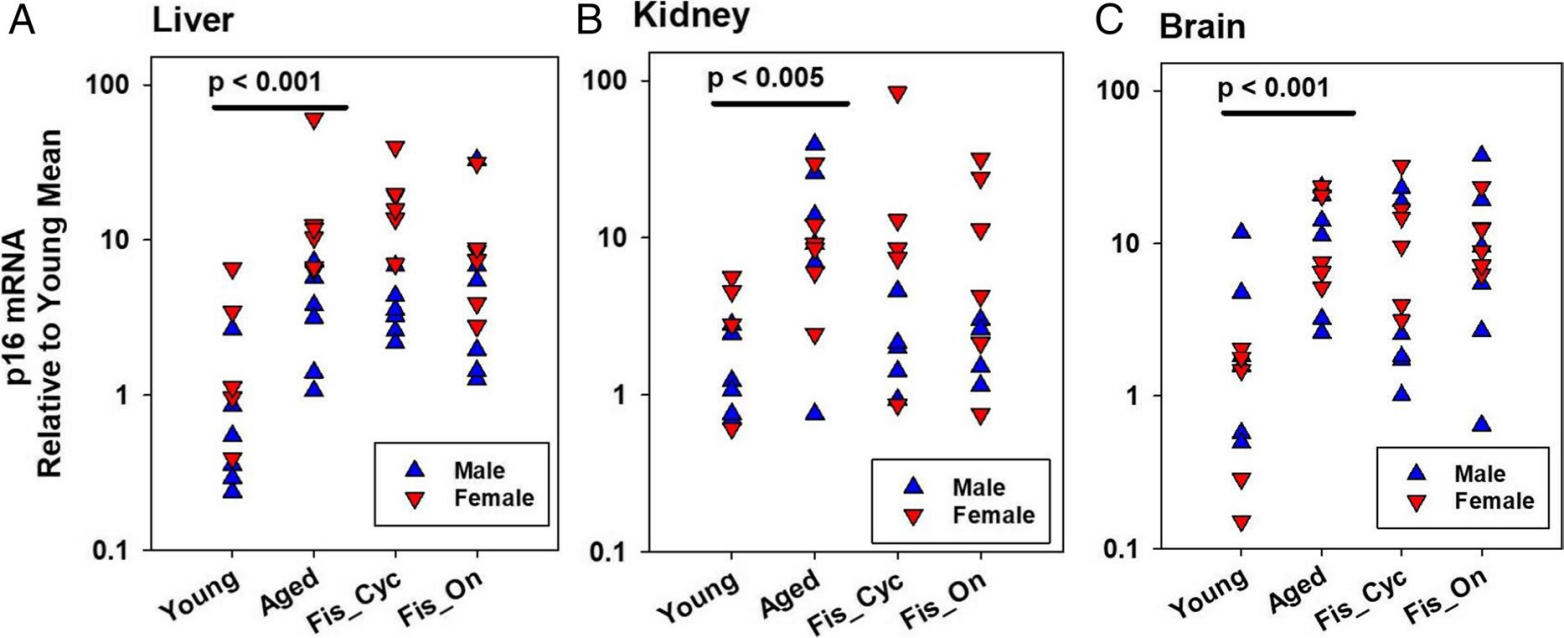
The p16 mRNA senescent cell marker increases with age in HET3 mice, but no senescent cell markers are reduced by either fisetin diet

To see if percentages of senescent cells increase with age in the UM-HET3 mice, p16^Ink4a^ mRNA was compared in young and old controls and in old mice fed one or the other fisetin diet for 2–4 months. Aging led to significant increases in p16^Ink4a^ mRNA in the kidney, brain, and liver (Fig. 5A–C), but not p21^Cip1^, another marker of cellular senescence (data not shown). There was also no significant age-related increase in most SASP or inflammatory factors in the plasma (data not shown). Neither of the two groups of Fis-treated mice had significantly lower numbers of p16^Ink4a^-positive cells, compared to age-matched untreated control mice.

Diet containing fisetin at 600 ppm was fed from 20 to 22–24 months of age continuously = Fis On, and fed for 3 of 14 days = Fis Cyc. Tissues were taken when young controls were 6 months of age, while old controls and Fis fed mice were 22–24 months of age. In a two-factor ANOVA (sex, group, interaction), there were no significant interactions, so males and females were pooled in a one-factor ANOVA comparing the four treatment groups (Young, Aged, Fis_Cyc, Fis_On) shown in A –C. Units for the X-axis in A–C are cycle threshold (CT) = number of PCR cycles before a specific mRNA fluorescent signal is clearly above the background noise, but given relative to the mean value for the young control for that organ.

## Discussion

As found for 17-α-estradiol [3, 8], and Canagliflozin [11], significant lifespan benefits from Asta and Mec were seen only in males (Table 1). Female controls lived longer than male controls, as usual (Table 1). Asta increased male median lifespan at all 3 sites; only 2 sites benefited from Mec (Tables 2, 3, and 4). Body weights did not explain either set of lifespan benefits. It will be helpful, in follow-up work, to test different doses of these agents, because it is unknown whether the doses used in this initial study were optimal for extending lifespan.

Oxidative stress and chronic inflammation may play an important role in the pathogenesis of aging and age-related diseases [59, 60]. The Nrf2 inducer astaxanthin (Asta) is a promising longevity candidate due to its potent antioxidant and anti-inflammatory activity, favorable localization in mitochondria and other cellular membranes throughout the body, and excellent safety profile. This is the first study to evaluate Asta’s efficacy in a mammalian lifespan model and builds on previous studies demonstrating its lifespan benefits in model organisms and positive influences on mechanisms of aging. While a limited number of interventions, such as rapamycin, have shown efficacy in ITP studies, it is important to find agents with safety and tolerability profiles that support chronic clinical use, such as Asta, whose benefits may extend to cardiovascular [61, 62], neurological [63], and other forms of age-dependent disease [19].

MEC is an anti-histamine/anti-vertigo/anti-nausea medicine. It also has central anticholinergic actions. It was selected for this study because it binds mTOR and inhibits mTORC1 yet has fewer side effects than rapamycin. It will be important to test if the reduction of mTORC1 function is responsible for Mec-mediated lifespan extension in UM-HET3 males. There are two problems with this hypothesis. First, the benefits of Mec, at the dose used are only about half those of rapamycin [5, 10]. Tests of higher doses are now in progress. Second, rapamycin increases lifespan in females more effectively than it does in males, while Mec in the current study failed to benefit females significantly. This may be because blood levels of Mec were lower in females, as noted in the “Results” section. This sex difference may provide interesting information.

We started Fis at 20 months, following Yousefzadeh et al. [44], who started at 85 weeks (about 20 months) of age; the Yousefzadeh study followed mice that were offspring of the cross between C57BL/6 with FVB mice. Their diet contained 500 ppm Fis and extended both median and maximal lifespan, but numbers were small. Our diet contained 600 ppm Fis, a slight increase. In our study, some mice received Fis continuously, and others received it over 3-day periods every 14 days, as opposed to rapid administration by oral gavage. In both cases, Fis did not significantly affect lifespan (Table 1, Fig. 3). Mice euthanized at 22–24 months, but otherwise treated as lifespan mice, were fed Fis on each of those schedules for 2–4 months. Two months should be sufficient to remove senescent cells, as Yousefzadeh et al. [44] (Fig. 2C), using in vivo mouse markers, found highly significant senescent cell reduction immediately, 2 weeks, and 4 weeks after feeding 500 ppm Fis.

Figure 5 shows that Fis, using the doses and route described here, did not significantly lower the amount of p16^Ink4a^ mRNA in UM-HET3 mouse liver, kidney, or brain. p16^Ink4a^ whole tissue mRNA is one marker of senescent cell burden, but it is not a fully sensitive marker of senescence, for example, it is also expressed in other cell types such as activated macrophages [64]. The comparison between young and aged control mice confirmed the expected age-dependent rise in p16 cells in all three organs, but not p21^Cip1^ nor several other factors in tissue and in the plasma (data not shown). We had hoped that Fis would deplete senescent cells and thus test the important idea that the removal of senescent cells would lead to longer lifespan, but the absence of an effect on p16^Ink4a^-positive cells and the lack of inflammatory p21^Cip1+^ cells in older UM-HET3 mice prevented us from addressing this question. Also, we were not able to reproducibly detect Fis in the plasma of the treated mice. It may be possible in future protocols to use approaches, such as bolus gavage of Fis or use of other senolytic agents, to provide better information about the potential role of senescent cells in mouse aging. The basis for the discrepancy between our lifespan results and those of Yousefzadeh et al. [44] is probably not the source of drug, dose, and route of Fis administration. Our study is more adequately powered, using 30 × larger numbers of mice compared to the earlier study. The discrepancy may reflect the use of different mouse stocks or inter-laboratory variations in husbandry conditions. Further studies to analyze the types and location of senescent cells that might increase with age in UM-HET3 mice and how they differ from other mouse models in regard to their upregulated senescent cell anti-apoptotic pathways (SCAPs), as well as the use of Fis and other senolytic agents by gavage, might help to clarify these issues.

Each drug (except SG1002, which could not be assayed using our methods) was detectable in food pellets, and in many cases in plasma of treated mice as detailed in the “Experimental procedures” section. We purchase new diet preparations every 4 months, and amounts of three of the six interventions—Fis, Mec, and DMF—tested in each independent diet preparation were lower and much more variable than expected. The reason for this is not known. This illustrates the importance of testing actual amounts of each intervention in each independent preparation of the diet.

Asta and DMF are both Nrf2 inducers; while both had low concentrations sometimes in the diet, we used about 30 times more Asta, which may explain why it increased the lifespan in males while DMF had no effect. Plasma measures of Fis were not reliable, so we do not know if plasma Fis was detectible in mice continuously fed Fis-containing chow. We performed no PK studies on UM-HET3 mice, so details of drug metabolism were not defined. In general, it is possible that any of these drugs might have led to health benefits if used at a different dose, in another stock of mice, administered differently, or at different ages. However, the doses and schedules used for each of the drugs were optimized to retard aging based on the current literature. Thus, a possible interpretation is that none of the other drugs tested in C2018 or C2019 slows aging or prevents disease in a genetically heterogeneous mouse population under highly sheltered constant conditions designed to minimize stress.

New positive results (here with Asta and Mec) give us new tools to help understand methods to retard damage from aging and evaluate mechanisms that cause such damage. For example, Miller et al. [65] reviews a series of papers, using drug treatments that increase lifespan (rapamycin, acarbose, 17-α-estradiol, and canagliflozin), and genetic models that increase lifespan (Snell, Ames, PAPPA-KO, and GHRKO mice), plus diet restriction groups. Results suggest shared physiological and molecular traits found in all nine varieties of these slow-aging mice, and which therefore suggest pathways that an agent must affect to delay mortality in laboratory mice. This kind of work has clinical potential, to suggest techniques that retard mammalian aging.

Lifespan studies done by the ITP demonstrate that, when following precise standards, mouse lifespan can be reproducibly extended by test agents in the diet [2, 3, 5–7, 9–11, 66]. Positive results, like those reported here for Asta and Mec, are important contributions to the literature, providing new clues about mechanisms of aging and pathways to prevent age-related disease and disability. As more agents increasing lifespan and otherwise retarding aging are defined, theories about how aging is retarded will be tested and new understandings will emerge. Ultimately, this will suggest clinical treatments.

## Experimental procedures

### Mouse production, maintenance, and estimation of lifespan

UM-HET3 mice were produced at each of the three test sites as previously described [2, 5, 9], where environmental conditions are presented in detail. The dams of the test mice were CByB6F1/J, JAX stock #100,009 (dams, BALB/cByJ; sires, C57BL/6 J). The sires of the test mice were C3D2F1/J, JAX stock #100,004 (dams, C3H/HeJ; sires, DBA/2 J). In each site, breeding mice were fed LabDiet® 5008 mouse chow (PMI Nutritional International, Bentwood, MO). As soon as mice were weaned, they were fed LabDiet® 5LG6 distributed to the test sites from the same batch. Males were initially housed 3 per cage, while females were housed 4 per cage; numbers per cage declined as mice died. Figure 4 shows that lifespans for both female and male controls were reasonably similar at all 3 sites.

Details of the methods used for health monitoring were provided previously [2, 5, 9]. In brief, each of the three colonies was evaluated four to twelve times each year for infectious agents dangerous to mice. All such surveillance tests were negative for pathogens at all three sites throughout the entire study period.

### Removal of mice from the longevity population

Mice were removed from the study because of fighting or accidental death (for example, during chip implantation) or chip failure, or because they were used for another experimental purpose. For log-rank survival analyses, all such mice were “censored,” i.e., treated as alive at the date of their removal from the protocol and lost to follow-up thereafter. These mice were not included in calculations of median lifespan. For the mice in Tables 1 and 2, the numbers removed were C2018 males: 3 at JAX, 42 at UM, 7 at UT; C2018 females: 0 at JAX, 6 at UM, 11 at UT; C2019 males: 23 at JAX, 26 at UM, 4 at UT; C2019 females: 0 at JAX, 7 at UM, 6 at UT. Most of the males were removed because of fighting, and no mice were removed for experimental purposes.

The statistical approach used is the Kaplan–Meier calculation, based on the log-rank test, in which mice that do not die a natural death (typically because of removal for humane reasons) are treated as known to be alive at the data of removal, with unknown dates of death.

### Estimation of age at death (lifespan)

At UM and UT, mice were examined daily for signs of ill health from the time they were set up in the experiment. At JAX, mice over 500 days of age were examined daily and twice a day once they were marked as ill. Mice were euthanized for humane reasons if so severely moribund that they were considered, by an experienced technician, unlikely to survive for more than an additional 48 h. A useful endpoint criterion is the non-responsiveness of a mouse to being touched, and which is usually accompanied by one or more of the following: slow respiration, feeling cold to the touch, a hunched-up appearance with matted fur, signs of sudden weight loss, failure to eat and drink, prominent appearing ribs and spine, and sunken hips. The age at which a moribund mouse was euthanized was taken as the best available estimate of its natural lifespan. Mice found dead were also noted at each daily inspection, giving the lifespan.

### Control and experimental diets

TestDiet®, a division of Purina Mills (Richmond, IN), prepared batches of radiation-sterilized LabDiet® 5LG6 food that were ground to powder, and to which each test substance was added, as well as control diets from the same large batch treated the same way except that no test agents were added when diets were ground and mixed. These were prepared at intervals of approximately 4 months, and TestDiet® shipped food from the same batch to each of the three sites. For the interventions in this paper, the doses used were suggested by the experts who proposed the compound. The ITP then performed pilot studies to be sure that the mice were not harmed over 8 weeks. To assay actual amounts of intervention in each preparation, 3 diet pellets from different parts of the diet preparation box or from different boxes of the same preparation were collected. From each pellet, a 90-mg sample from each end and the middle were removed. These nine equal samples from all three pellets were pooled. Amounts of intervention were measured in three samples from the pooled mixture. The average of those three samples measured the actual percentage of the target amount in each diet preparation.

### Test agents given to cohort 2018

- (a) Fisetin (Fis) was purchased from Bioriginal (Anaheim, CA). Fis was started at 20 months and either fed continuously (Fis_On) or a repeated cycle of 3 days on and 11 days off (Fis_Cyc). Both groups were fed at a target amount of 600 ppm in the diet. Fis was quantified in mouse food by HPLC/UV, with the Fis peak area for each unknown sample compared against a linear regression of calibrator peak areas. In plasma, Fis was quantified by LC/MS/MS. Ratios of Fis, geraldol, and kaempferol peak areas to lidocaine D10 peak areas for each unknown sample were compared against a linear regression of the ratios obtained by the calibration samples to quantify each drug. Percentages of target amounts in diet samples received are given, followed by the date (month/year): 112 (7/18) in the pilot study diet, and 109 (1/20), 120 (5/20), 85 (9/20), 111 (3/21), and 69 (7/21) so mean% ± SE (*n*) = 99 ± 9 (5) in the lifespan study diet. Tests of Fis in plasma were not reliable.
  Assays for senescent cells were performed by the Robbins lab, using their standard techniques. Tissues came from mice fed 600 ppm fisetin starting at 20 months of age and continuing for between 8 and 16 weeks. These mice were euthanized between 8 a.m. and 10 a.m. On each day, at least one animal from each group was euthanized, although only one sex per day. Because of the number of tissues taken, only a maximum of 6 mice were euthanized on a single day. After blood collection and euthanasia by cervical dislocation, liver tissue was aliquoted into 6 cryo tubes and frozen in liquid nitrogen, then stored at − 80 °C.
- (b) SG1002, a hydrogen sulfide donor, was purchased from Sulfagenix (Oro Valley, AZ) and fed at a target amount of 240 ppm in the diet. Mice were fed this diet continuously starting at 18 months of age in C2018 and at 5–9 months of age in C2019. This material could not be assayed using the methods available. This S[n] molecule is not well detected using older methods like UV, IR, or EC and even shatters into large numbers of fragment peaks in MS.

### Test agents given to cohort 2019

- (c) Astaxanthin (Asta) was obtained from Cardax (Honolulu, HI) and fed at a target amount of 4000 ppm in the diet. The Asta supplied by Cardax consisted of synthetic Asta finely dispersed in a water dispersible beadlet formulation. The superior bioavailability of synthetic Asta beadlets compared to microalgal Asta was demonstrated in a human pharmacokinetic study (approximately threefold higher exposure in plasma over 24 h; [66] and in a non-human primate pharmacokinetic study (approximately eightfold higher exposure in plasma over 96 h; [67]). Importantly, synthetic Asta is a pure form of Asta, whereas microalgal and other natural extracts of Asta are complex mixtures of Asta esters (mono-esterified and di-esterified with fatty acids), Asta, and other byproducts [68–70]. Synthetic Asta as utilized herein is comprised of the following stereoisomers: (3*S*,3′*S*), (3*R*,3′*R*), (3*S*,3′*R*), and (3*R*,3′*S*), all of which are naturally occurring [71]. Synthetic Asta has demonstrated efficacy across a range of human, animal, and cell culture studies, for example [72–74], and its excellent safety profile has been well established in rigorous toxicity studies [20, 75]. Synthetic Asta beadlets are also utilized in the following commercially available dietary supplements: ZanthoSyn® (Cardax, Honolulu, HI) and AX3™ (AX3 Life, Honolulu, HI).
  The concentration of Asta in each batch of chow prepared for this study was initially quantified using LC/MS. Percentages of target amounts in diet samples received are given, followed by the date (month/year): 183 (2/20) in the pilot study diet, and 64 (9/20), 11 (12/20), 14 (4/21), 11 (7/21), 13 (9/21), 32 (1/22), 104 (2/22), 101 (4/22), and 66 (9/22) so mean% ± SE (*n*) = 46 ± 13 (9) in the lifespan study diet. These highly variable analytical results, averaging less than half the target amount, were unexpected given the uniform supply of Asta and consistent method of chow preparation. Samples of each batch were retained and will be reanalyzed. Nevertheless, even with the uncertainty regarding the actual versus target concentration of Asta in the chow, Asta showed a significant extension of lifespan in the study.
- (b) Meclizine (Mec) was obtained from Pfizer (New York, NY) and fed at a target amount of 800 ppm in the diet. The pure standard, Mec, and the internal standard, clonazepam, were obtained from Sigma Aldrich (St. Louis, MO). Mec and clonazepam were eluted with a gradient and quantified using the HPLC/UV. Percentages of target amounts in diet samples received are given, followed by the date (month/year): 99 (8/19), 72 (10/19), and 79 (2/20) in the pilot study diet, and 33 (5/20), 77 (10/20), 75 (12/20), 63 (4/21), 76 (7/21), 93 (9/21), 74 (1/22), 63 (4/22), and 56 (9/22) so mean% ± SE (*n*) = 68 ± 6 (9) in the lifespan study diet. Amounts of Mec were low in many cases. Since Mec is an emulsion of crushed tablets, sources of variability include tablet crushing, Mec attaching to inert constituents of the tablet, and the emulsion, really a suspension which can settle. We asked TestDiet® to shake well before adding, and we only shipped overnight the day before they had the floor ready to make this formulation. In plasma, Mec in serum was quantified using LC/MS/MS. After mixing with a known amount of lidocaine, the ratio of Mec peak area to lidocaine D10 peak area for each unknown sample was compared against a linear regression of the ratios obtained by the calibration samples to quantify Mec. Amounts in serum given as minimum, maximum, and average (month/year measured) were 12, 139, and 88 ng/ml in males (10/19) and 8, 31, and 22 ng/ml in females (10/19) in the pilot study, and 92, 298, and 175 ng/ml in males (4/20) and 25, 268, and 115 ng/ml in females (4/20) in the main study, respectively. More Mec was found in male serum, although amounts varied greatly. This may help explain why only males had increased lifespans.
- (c) Dimethyl fumarate (DMF) was obtained from Cayman Chemical (Ann Arbor, MI) and fed at a target amount of 120 ppm in the diet. Standards for analysis were obtained from Cayman Chemical. In the HPLC/UV, peak areas for each unknown sample were compared against a linear regression of known DMF in diet calibrator peak areas to quantify DMF. Percentages of target amounts in diet samples received are given, followed by the date (month/year): 10 (8/19) and 29 (10/19) in the pilot study diet, and 20 (2/20), 117 (5/20), 25 (11/20), 13 (2/21), 34 (5/22), and 1 (9/22) so mean% ± SE (*n*) = 35 ± 17 (6) in the lifespan study diet. Amounts in diet were low except at 5/20. As noted above, this may help to explain the absence of lifespan effects. DMF in serum was quantified using LC/MS/MS with known amounts of internal standard, DMF D2. The ratio of DMF peak area to DMF D2 peak area for each unknown sample was compared against a linear regression of the ratios obtained by the calibration samples to quantify DMF. Amounts in serum given as minimum, maximum, and average (month/year measured) were 14, 15, and 15 ng/ml in males (11/19) and 15, 27, and 21 ng/ml in females (11/19) in the pilot study, and 5, 7, and 6 ng/ml in males (7/20) and only one result in females of 6 ng/ml (6/20) in the main study, respectively.
- (d) Mycophenolic acid (MPA) was obtained from MilliporeSigma (Burlington, MA) and fed at a target amount of 6.7 ppm in the diet. The standard MPA and the internal standard mycophenolic acid D3 (MPA D3) were obtained from Sigma-Aldrich (St. Louis, MO). The ratio of MPA peak area to MPA D3 peak area for each unknown sample was compared against a linear regression of the ratios obtained by the calibration samples to quantify MPA by HPLC and MS. Percentages of target amounts in diet samples received are given, followed by the date (month/year): 84 (8/19) in the pilot study diet, and 83 (1/20), 68 (5/20), 82 (11/20), 78 (2/21), 94 (6/21), 112 (7/21), 100 (9/21), 131 (5/22), and 75 (9/22) so mean% ± SE (*n*) = 91 ± 7 (9) in the lifespan study diet. Using a similar technique, amounts in serum given as ng/ml and listed as minimum, maximum, and average (month/year measured) were males: 45, 83, 64 (11/19); females: 38, 164, 101 (11/19). Amounts of MPA in diets were similar to those expected, and measured blood levels were significantly above negative controls.
- (e) 4-Phenylbutyrate (PBA) was obtained from Cayman Chemical (Ann Arbor, MI) and fed at a target amount of 1000 ppm in the diet. The standard PBA for quantitation was obtained from Cayman Chemical. Analyzed with HPLC and a UV detector, peak areas for each unknown sample were compared against a linear regression of calibrator peak areas to quantify PBA. Percentages of target amounts in diet samples received are given, followed by the date (month/year): 17 (5/19) and 92 (8/19) in the pilot study diet, and 101 (1/20), 109 (5/20), 118 (11/20), 105 (2/21), 106 (6/21), 86 (7/21), 93 (9/21), 150 (5/22), and 97 (9/22) so mean% ± SE (*n*) = 107 ± 6 (9) in the lifespan study diet. Plasma was analyzed using LC/MS/MS. The ratios of 4-PB, PAA and PLG peak areas to 4-PB D11 peak areas for each unknown sample were compared against a linear regression of the ratios obtained by the calibration samples to quantify each drug. Amounts in serum given as ng/ml and listed as minimum, maximum, and average (month/year measured) were 33, 433, and 233 in males (11/19) and 200, 512, and 356 in females (11/19), respectively. Amounts of PBA were similar to those expected in the diets, and it was present in blood.

### Statistical methods

Significance tests about survival effects are based upon the two-tailed log-rank test at *p* < 0.05, stratified by the test site, with censored mice included up until their date of removal from the longevity population. Data from male and female mice are considered separately. In statistical tests of lifespan differences described in the text,* p*-values are two-tailed and reported without adjustment for multiple comparisons. Statistical claims related to maximum lifespan are based on the Wang–Allison test [76] using Fisher’s exact test to compare the proportions of surviving mice, in control and test groups, at the age corresponding to the 90th percentile for survival in the joint distribution of the control and test groups. For the pooled data sets, surviving mice were enumerated at the 90th percentile age for each site separately, and these counts were combined for the overall Fisher’s exact test.

The raw data used here are available at the Mouse Phenome Database https://phenome.jax.org/projects/ITP1, The Jackson Laboratory, Bar Harbor, ME. This is supported by the Mouse Phenome Project and is a public data repository that provides the authoritative source for the raw and summary data from the ITP, along with visualizations for exploration of lifespan and related phenotype data.

## Data Availability

Mouse Phenome Database https://phenome.jax.org/projects/ITP1, The Jackson Laboratory, Bar Harbor, ME.
